# A common framework for the analysis of complex motion? Standstill and capture illusions

**DOI:** 10.3389/fnhum.2014.00999

**Published:** 2014-12-18

**Authors:** Max R. Dürsteler

**Affiliations:** Vestibulo-Oculomotor Lab., Department of Neurology, University Hospital ZürichZürich, Switzerland

**Keywords:** complex motion, rotation, expansion, color vision, stereo vision, motion transparency, motion illusion

## Abstract

A series of illusions was created by presenting stimuli, which consisted of two overlapping surfaces each defined by textures of independent visual features (i.e., modulation of luminance, color, depth, etc.). When presented concurrently with a stationary 2-D luminance texture, observers often fail to perceive the motion of an overlapping stereoscopically defined depth-texture. This *illusory motion standstill* arises due to a failure to represent two independent surfaces (one for luminance and one for depth textures) and *motion transparency* (the ability to perceive motion of both surfaces simultaneously). Instead the stimulus is represented as a single non-transparent surface taking on the stationary nature of the luminance-defined texture. By contrast, if it is the 2D-luminance defined texture that is in motion, observers often perceive the stationary depth texture as also moving. In this latter case, the failure to represent the motion transparency of the two textures gives rise to *illusionary motion capture*. Our past work demonstrated that the illusions of motion standstill and motion capture can occur for depth-textures that are rotating, or expanding / contracting, or else spiraling. Here I extend these findings to include stereo-shearing. More importantly, it is the motion (or lack thereof) of the luminance texture that determines how the motion of the depth will be perceived. This observation is strongly in favor of a single pathway for complex motion that operates on luminance-defines texture motion signals only. In addition, these complex motion illusions arise with chromatically-defined textures with smooth transitions between their colors. This suggests that in respect to color motion perception the complex motions' pathway is only able to accurately process signals from isoluminant colored textures with sharp transitions between colors, and/or moving at high speeds, which is conceivable if it relies on inputs from a hypothetical dual opponent color pathway.

## Introduction

Imagine walking with your dog during heavy snow fall. While your dog is running across a meadow covered with snow you are able to perceive both, the movements of the dog and motion of the falling snow flakes between yourself and the dog. Else imagine walking in a forest, while a deer is passing in a small clearing behind trees whose branches are wiggling in the wind. You're able to perceive simultaneously the movement of the deer and those of the branches. In both scenes, one perceives overlapping visual stimuli moving in different directions, i.e., one experiences *motion transparency*. Motion transparency is defined as the capacity to perceive simultaneously the motion of two sets of stimuli that are moving at different speeds and/or in different directions on physically overlapping surfaces (for a review see Snowden and Verstraten, 1999). Considering the examples above, snowflakes or branches are defining an upper surface like a window grille, through which we look at the underlying surface of the dog or the deer.

A dog, a deer, snowflakes or branches are composite visual stimuli containing luminance, color and depth information. In this paper we are interested in which of the different visual submodalities like luminance, color, or depth contribute to the perception of motion transparency. We will look primarily at the interactions of luminance and color or depth stimuli.

In our color and luminance illusions, a Julesz (1960) type random dot pattern is used, where half of the pixels are black opaque and half transparent. One can think of the random dot texture as an overlying surface forming a window grille through which one looks at the underlying surface with luminance information (cf. accompanying video “Motion Transparency and Flicker with Luminance Textures”) or color stimuli (video “Motion Transparency for Luminance and Color”). In all of these introductory videos, translating luminance, color or stereo gratings and luminance textures move the same speed (256 Pixels/s or 1 cycle/s for the gratings). The luminance grille overlying the color stimuli relaxes the requirement for the different colors to be strictly isoluminant by blocking most of the residual luminance content in the color stimuli.

The current stereo illusions are created with the same type of random dot pattern with equal number of black opaque and white opaque dots (cf. video “Motion Transparency for Luminance and Depth”). The motion of the random dot pattern itself can be manipulated independently from the horizontal binocular disparities encoded in the patterns for the left and right eye to create the stereo stimuli. One can think of the random dot pattern used to encode the disparities as the window grille through which we look at the depth stimuli.

For the illusions, two sets of stimuli are shown on independent overlapping surfaces. To keeps things simple, only stimuli of one of the overlapping surfaces are actually moving: either the luminance texture in the overlying surface is stationary, while the luminance, color or depth stimuli of the underlying surface are moving (think of it as looking through the branches at the running deer while there is no wind) or the luminance texture is moving while the luminance, color or depth stimuli of the underlying surface are stationary (searching during a heavy snowfall for the dog, while he is standing still).

To begin with let's consider the experimental setup, where the overlying luminance texture is stationary (cf. left panel in the introductory videos). We will pose the question, if we can perceive the motion of the underlying physically moving stimuli. If the answer is no, we perceive motion standstill. “*Motion standstill*” (Lu et al., 1999b) is the failure to perceive the motion of underlying stimuli seen through a stationary grille, while the structure of the stimulus is still clearly visible. You may think of getting just some glimpses of an apparently stationary deer through the stationary branches while it is actually running. While this seems unlikely for a moving composite visual target such as a deer, moving targets which are only defined by color or depth differences, may be more difficult to track underneath a surface defined by luminance textures. For example, look at the left panel in the videos about color or stereo vision. You need to follow very attentively the color stripes or depth ridges to perceive their motion. According to previous research, there is a difference between either luminance or color and stereo motion perception. The motion perception of a luminance target is mediated by an efficient velocity based, first order motion system (see Cavanagh and Mather, 1989), whereas the motion perception of targets defined by color differences (Lu et al., 1999b) or depth (Lu and Sperling, 1995b, 2001) only are believed to depend on a less sensitive and less efficient salient feature tracking system.

Then let's consider the second experimental setup (cf. right panel in the introductory videos), where the overlying luminance texture is moving. Here we pose the question, if we perceive the underlying physically stationary stimuli as not moving with the overlying stimuli. If the answer is no, we perceive motion capture. “*Motion capture*” (Murakami and Shimojo, 1993) is the illusory percept where one perceives a physically stationary stimulus as moving together with the moving grille. Imagine perceiving a dog falling down together with the snowflakes, while he is actually seated. While it is absurd for a composite visual target such as a dog, a stationary target defined by color or depth differences may only appear to slip together with the random dot texture moving in the overlying surface. E.g., look at the right panels in the videos about color or stereo vision. Here random dots are sliding over physically stationary color stripes or stereo ridges. You need to fixate stripes or ridges attentively to escape the percept that they are sliding in the direction of the dots.

Both motion standstill and motion capture are examples of a failure of motion transparency. We have not yet looked at examples where both the grille and the underlying stimulus are moving (Adelson and Movshon, 1982; Hibbard and Bradshaw, 1999). Note that the above definitions of motion standstill and motion capture are adapted to the context of motion transparency. Their original definitions are: *In “motion standstill” a physically quick moving object appears stationary, while its details are clearly visible* (Lu et al., 1999b). *“Motion capture” is known as a phenomenon in which the percept of a physically stationary stimulus (the target) is influenced by an adjoining moving stimulus (the inducer) to move in the same direction as the inducer* (Murakami and Shimojo, 1993).

A well-known technique introduced by Julesz (1960) to avoid most blocking or capturing effects of a (stationary or moving) grille on the motion of the underlying stimulus, is the use of a *dynamic* or *flickering* grille, where the grille's random-dot pattern is renewed at every frame change. With such a dynamic noise mask, motions of the underlying color or stereoscopic stimuli and the luminance mask are unbalanced at a local level thus providing the preconditions for motion transparency (Qian et al., 1994a). When a dynamic luminance random-dot pattern is used as a grille, there is no coherent common luminance motion vector or zero motion as when using a moving or a stationary random-dot grille. Thus, coherent motion of the underlying stimulus may be easier to perceive, as long as it is moving at supra-thresholds speeds for motion transparency (cf. last two sections in the videos cited above). A dynamic noise mask can be used to block off minor luminance contaminations (Baker et al., 1998; Michna and Mullen, 2008).

Previous studies of motion transparency used luminance (Qian et al., 1994a,b; Edwards and Greenwood, 2005), color (Krauskopf and Farell, 1990; Cropper et al., 1996; Krauskopf et al., 1996) and depth (Chang, 1990) stimuli. They were focused on translational motion but not complex *motion* such as *rotation, expansion/contraction* or *pure shearing* (Koenderink, 1986). All these works look at a peculiar modality, which could influence motion transparency perception. How the submodalities mentioned above interact with each other could shed a light on how the brain processes these kinds of information. To answer to this puzzling question, I looked primarily at the interactions of two modalities, namely luminance, and either color or depth, by producing illusions, i.e., visual percepts that the brain cannot accurately deal with. As mentioned above, chromatic and stereoscopic motion perception is mediated by a feature tracking system. For luminance-based complex motion stimuli, detection of complex motion requires a two-stage process (Morrone et al., 1995) with the anatomical substrate for the second stage thought to be in MSTd (Saito et al., 1986; Tanaka and Saito, 1989; Duffy and Wurtz, 1991a,b). Since attentional factors are important in the determination of saliency (Lu and Sperling, 1995a), it is unlikely that the salient feature system feeds into a hierarchical higher complex motion processing stage. Thus, a feature tracking based motion system should fail to perceive complex motion. The capacity to detect complex motion may be restricted to general-purpose first order luminance motion mechanism with output to a specialized complex motion processing stage. If there is no complex motion processing stage for complex depth or color motion, we should find both complex motion stereo and color standstill illusions when pitting luminance against depth or color motion.

In fact, Dürsteler and Lorincz (2014) demonstrated stereo standstill illusions for rotation, contraction/expansion and spiraling motion; here, stereo shear standstill illusions will be shown.

I then expand our observations of complex motion detection in stereovision to color vision to look for failures to discriminate complex color motion. The hypothesis that we are unable to perceive complex color motion appears to run against expectation. As children, we may have played with color wheels and we never had any trouble to perceive them rotate below the speeds where colors start mixing. For a demonstration of a chromatic deficit, the different colors of the color wheel have to be exactly isoluminant with smooth transitions between different colors both for physical reasons (avoidance of chromatic aberrations) and for reasons of, how color signals are processed in the visual pathway (see Discussion). Deficiencies in perceiving color rotation has been described by Cavanagh (1992). Demonstrating complex color motion standstill and capture illusions adds evidence to our hypothesis that we are blind for complex color motion.

In *conclusion*, my main hypothesis is that the putative feature tracking or third order motion system as used in stereo and color vision cannot recognize complex motion without the help of supplementary motion system like the first order luminance motion system. To demonstrate this point, we will use a complex motion transparency paradigm pitting luminance against stereo or color motion, where a failure of complex motion transparency will be taken as evidence against the existence of luminance-independent complex motion detectors in the stereo or the color system.

## Description of complex motion illusions

### Complex motion illusions in stereo vision

The complex motion illusions described below are seen best when fixating at the center of rotation, contraction/expansion or pure shear. It is important to note that in all of these illusions, local (translational) motion can still be well perceived, when fixating or tracking a local feature near the periphery of the stimulus. Table 1 lists the motion parameters used in preparing the accompanying videos.

**Table 1:** **Stimulus parameters used in demonstration videos**.

**Paradigm**	**Rotational speed [°/s]**	
	**Sector wheel**	**Random-dot texture**
Stereo rotation standstill	12	0
Stereo rotation	12	12
Stereo rotation capture	0	12
Color rotation standstill	6 or 24	0
Color rotation	6	6
Color rotation capture	0	6 or 24
Color border rotation standstill	4	0
Color border rotation	4	4
Color border capture	0	4
Contrast modulated rotation standstill	4	0
Contrast modulated rotation	4	4
Contrast modulated rotation capture	0	4
Luminance rotation standstill	4	0
Luminance rotation	4	4
Luminance capture	0	4
Luminance rotation standstill	4	0
Luminance rotation	4	4
Luminance capture	0	4
**Paradigm**	**Scaling factor [%]**	
	**Ring wheel**	**Random-dot texture**
Stereo scaling standstill	117.6	100
Stereo scaling	117.6	117.6
Stereo scaling capture	100	117.6
Color scaling standstill	107.9	100
Color scaling	107.9	107.9
Color scaling capture	100	107.9
Contrast modulated scaling standstill	103.9	100
Contrast modulated scaling	103.9	103.9
Contrast modulated scaling capture	100	1.039
Stereo shearing standstill expansion	120	100
Stereo shearing standstill compression	83	100
Stereo shearing capture expansion	100	120
Stereo shearing capture compression	100	83
Color shearing standstill expansion	108.2	100
Color shearing standstill compression	92.4	100
Color shearing capture expansion	100	108.2
Color shearing capture compression	100	92.4

*Scaling factors in the table above are given for a period of 1 s. Please note, that scaling factors for a given time period increase as a power function of time. To obtain the total shearing of the color rings for 5 s take 1.079^5^ = 1.46 or 146%. In the stereo scaling application, the temporal frequencies obtained with the scaling factors above was 0.4 cycles (rings)/s, in the color scaling paradigm, the equivalent temporal frequency was 0.2 cycles/s*.

The illusions described below demonstrate a failure of the stereo system to detect rotating, scaling or shearing motions on its own. From a failure to detect rotation, expansion/contraction and shear, the basic components of optic *retinal* flow (Koenderink, 1986), such deficiencies of the stereo system should also exist for combinations of optic flow components. For instance, spiraling motion is a combination of expansion/contraction and rotation where clear deficits are found (Dürsteler and Lorincz, 2014).

It should be noted, that here we are concerned with the two-dimensional motion only, as it arises from the projection of a 3-D environment on the retina, and not on its three-dimensional interpretation (motion in depth in the cases of expansion/contraction or spiraling motion).

#### Methods

To develop an interactive animated web application demonstrating stereo motion illusions, the Microsoft Silverlight programing environment was chosen as it allows using pixel-shaders in web applications (Dürsteler and Lorincz, 2014). Good alternatives to Silverlight are game engines with a pixel-shader making facility like “Unity,” which was used in the preparation for the introductory videos to demonstrate motion transparency. A pixel-shader defines the color of a pixel (e.g., cyan, red, yellow, or black in an anaglyphic stereogram) as a function of its bitmap coordinates by default. Additional inputs can be constants (e.g., a maximal disparity) and additional bitmaps (e.g., a depth-map for producing a stereo anaglyph). Pixel-shader functions can be processed in parallel to speed up calculation time allowing one to produce animated red-cyan stereograms in real-time. Due to computing time and memory problems, the application had to imitate “flicker” by irregularly shifting, rotating, and scaling the same random-dot texture on each frame. The screen-capture facility of Microsoft Expression Encoder 4 allows recording from a running Silverlight application. It was used to prepare the accompanying videos about stereo- and contrast-modulated motion phenomena. Magix Video deluxe 2014 was used to convert video from the Windows Media to the MP4-format (resolution 1280 × 720 pixels at 29.7 frames/s). Unity has a built-in facility to record series of screenshots, which were converted into a MP4-video by Adobe Premier and Magic Video.

An inherent problem of static random-dot displays is the occurrence of relative or shearing motion along the horizontal axis reflecting disparity changes during the motion of a 3-D structure. At high speeds, the horizontal shearing motion becomes quite noticeable and degrades the strength of the stereo standstill illusions described below. The form of our 3-D stimuli was tailored to minimize the perceptual effects of horizontal shearing motion by using radial form elements (sectors) for rotation, circumferential elements (rings) for expansion/contraction and spirals for a combination of rotation and scaling.

#### Stereo motion standstill, stereo motion capture and flicker

Stereo *motion standstill* illusions have been found to occur in stroboscopic translation and rotation (Julesz and Payne, 1968) and rapid translational motion (Tseng et al., 2006). These illusions were produced using dynamic random-dot stereograms of a three-dimensional grating. In the Tseng et al. (2006) experiment, a physically translating 3-D grating, which was perceived as clearly moving at temporal frequencies lower than 4 Hz, appeared motionless at higher temporal frequencies, while its structural details were still discernible up to a temporal frequency of 7 Hz. In our demonstrations, a rotating 3-D structure is perceived as standing still. Similarly, an expanding/contracting or a shearing 3-D structure appears as standing still (cf. **Figures 2A–E**).

**Figure 1:**
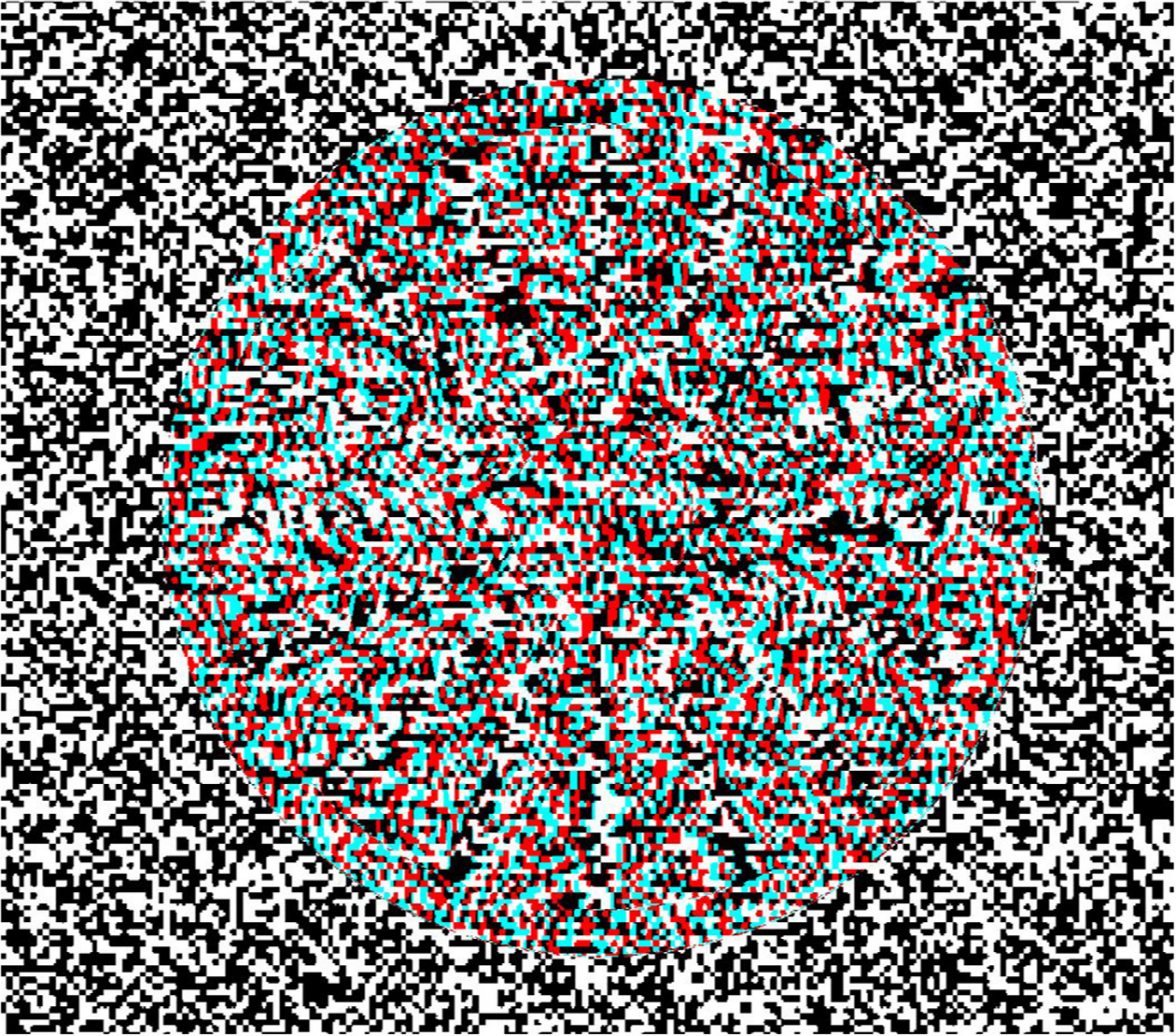
**Red-cyan anaglyph of a 3-D wheel**. You have to use red-cyan anaglyph glasses to see the figure in depth. The depth-profile of the wheel follows a sinusoidal function. This kind of wheel was used in the first public demonstration of the rotation standstill illusion (Dürsteler, 2008a,b).

In our demonstration, a rotating depth-encoding texture appears to induce its underlying stationary 3-D structure to co-rotate with it (cf. Figure 1). Similarly, an expanding/contracting texture leads to a perception of expansion/contraction, and a shearing texture to a perception of shearing of its physically stationary 3-D structure (cf. Figures 3A–D).

#### Stereo rotation standstill and capture illusion

**Figure 2:**
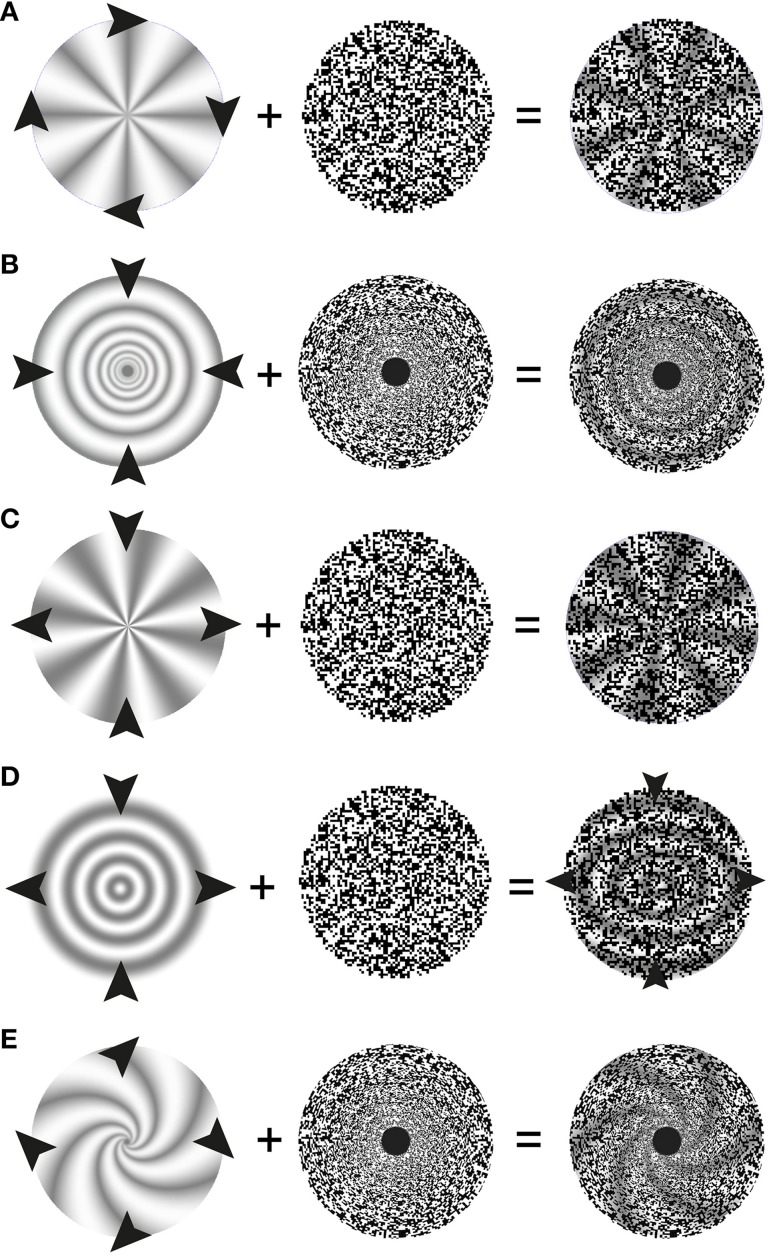
**Schematic description of stimulus elements and perceptual outcomes in stereo complex motion standstill illusions**. Arrow heads indicate the motion of the depth-map used in the present study. The middle column symbolizes the (stationary) random-dot texture used to encode the depth. The right hand column depicts the resulting paradoxical percepts: one perceives the 3-D as motionless; nevertheless they change their orientation over time. An exception are the shearing rings, where shearing is still perceived, albeit at a reduced speed. **(A)** Stereo rotation standstill. The arrowheads indicate a clockwise rotation of the sector wheel. The wheel's rotation is not perceived in the presence of a stationary random-dot pattern. To study scaling motion a different random-dot pattern was used where the size of texture pixels increases as a power function of eccentricity. **(B)** Stereo scaling standstill. The arrowheads indicate a contraction of the rings. **(C)** Stereo shearing standstill with sectors. The arrowheads indicate compression along the vertical axis of the sectors and an expansion along the horizontal axis. When the random-dot texture does not participate in shearing, the physically shearing 3-D sectors appear not to shear. **(D)** Stereo shearing slow-down with rings. The arrowheads in the middle indicate compression along the vertical axis of the rings and an expansion along the horizontal axis. The small arrowheads on the right indicate that some shearing motion of the rings is still perceived, albeit the overlying random-dot texture is stationary. **(E)** Stereo spiraling motion standstill. The arrowheads indicate a combination of a clockwise rotation and an expansion of a 3-D spiral (in reality, either a rotation or an expansion of the logarithmic spiral alone will achieve the same effect).

In the *stereo rotation standstill illusion* (SRSI), a rotating 3-D wheel is perceived as standing still. In the accompanying video “Stereo Rotation Illusion,” a 3-D wheel made out of eight convex sectors (cf. Figure 2A) is rotating at 12°/s. Using uncrossed disparities only rather than crossed disparities makes the motion more perspicuous (Phinney et al., 1994). When the overlying random-dot pattern is co-rotating, one perceives the 3-D wheel as rotating. However, when the dot pattern stops, the physically still rotating 3-D wheel appears as motionless. Yet while one does not perceive the whole wheel's rotation, one still clearly can track single features of the wheel like the peripheral end of a sector and follow it around the wheel's circumference. The illusion is quite robust: using the Silverlight application, a wheel rotating at more than 60°/s still appears as motionless. Even at this high speed, its structure remains visible. The main limiting factor is the occurrence of horizontal relative motion used to encode the disparity changes. In the video, there is a sequence where yellow markers sticking to the rotating wheel should inform the viewer of its physical speed. Note the paradoxical percept of rotating markers never sliding over the apparently stationary sectors. The occurrence of the SRSI does not depend on the use of a black and white random-dot texture to encode the depth of the 3-D wheel; any richly structured texture will do.

**Figure 3:**
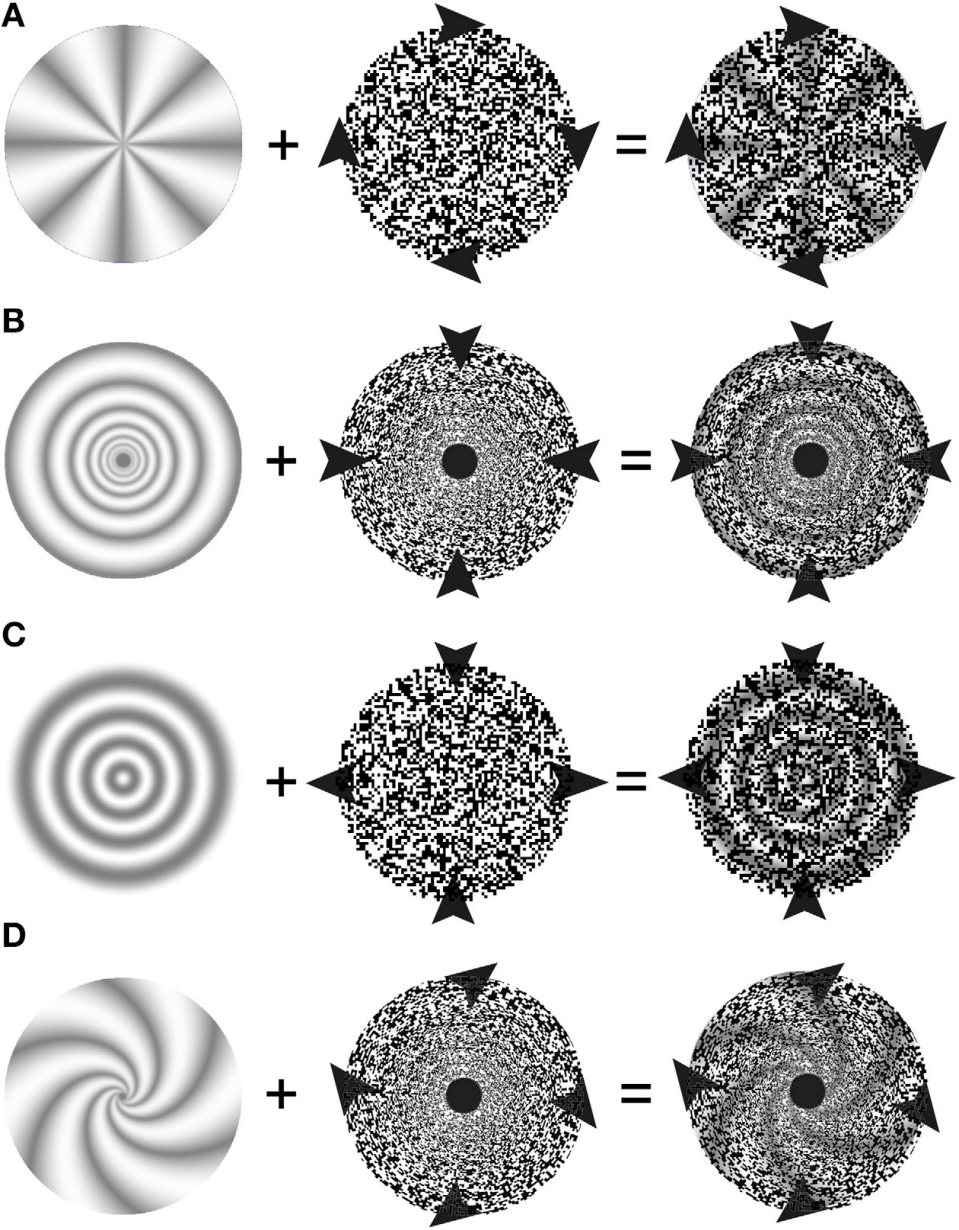
**Schematic description of stimulus elements and perceptual outcomes in stereo complex motion capture illusions**. The left hand column depicts the stationary depth-map. Arrowheads symbolize the motion of stimulus elements (here the random-dot patterns in the middle column) and of the perceptual outcomes in the right hand column: while the 3D-structures appear to move, they nevertheless do not change over time. **(A)** Stereo rotation capture: The arrows heads depict a clockwise rotation of the depth-encoding random-dot texture which leads to a percept of a joint rotation of the 3-D wheel and the random dots. **(B)** Stereo scaling capture. The arrowheads symbolize a contraction of the random-dot pattern that leads to a percept of contracting 3-D rings. A special random-dot texture, whose texture pixel sizes increases as a power function of eccentricity is used. **(C)** Stereo shearing capture. The random-dot texture is compressed along the vertical axis and elongated along the horizontal. There is a vivid percept of joint shearing of the underlying 3-D structure. **(D)** Stereo spiraling motion capture. Arrowheads depict an expanding and clockwise rotating spiraling motion of the random-dot pattern perceptually taking the 3-D spiral with it.

In the *stereo rotation capture illusion* (SRCI) demonstrated in the second part of the video “Stereo Rotation Illusion,” the rotation of the depth encoding texture captures the underlying 3-D wheel: the physically stopped 3-D wheel appears to co-rotate with the encoding random dot pattern. Even with stationary yellow markers this “*stereo complex motion capture illusion*” cannot be overcome. However, on local inspection of a single sector border or a ring, one perceives texture elements sliding over them. The motion capture effect is restricted to the part of the 3-D wheel underneath the rotating random-dot pattern. Making, for instance, the rotating texture smaller than the underlying 3-D wheel, induced the paradoxical percept of the inner part of the wheel rotating and the outer part stationary without breaking the wheel's spokes. The motion capture effect is so strong that it even can overcome counter-rotation of the 3-D wheel. Even the apparent center of rotation can be displaced, when the center of rotation of the encoding turning texture is shifted away from the center of rotation of the 3-D wheel (Dürsteler and Lorincz, 2014).

While using a dynamic (“flickering”) random dot display showing a rotating 3-D wheel, a percept of a jerky overall rotation may be achieved by directing one's attention to local features such as the rim of a sector. Unlike as with static random-dot stereograms, colored markers turning in synchrony with the underlying 3-D structure are now able to capture its rotation, leading to a percept of smooth complex rotation.

The strength of the SRSI and the SRCI depend on *shape factors*: to obtain a good SRSI the 3-D structure used has to be radially symmetric with more than 6 equally sized sub-elements. A rotating 3-D sinusoidal grid which was encoded using a stationary random-dot pattern is perceived as rotating, albeit very slowly with a jerky motion (cf. first part of the video “Stereo Grating Rotation Phenomena”). When compared to “real” rotation, where the grating and the encoding random-dot texture co-rotate, the ghost-like character of the perceived residual rotation becomes evident. To counter-balance the perceptual effects of horizontal relative local motion due to disparity changes during rotation, the grating is shown with its midpoint at the level of the display plane at zero disparity. Even steep depth gradients do not abolish the stereo standstill illusion: A rotating (12°/s) 3-D wheel made out of 12 flat sectors, whose depth was alternating between two depth levels, was still perceived as motionless.

The strength of the SRCI also depends on shape factors such as the radial symmetry of the 3-D structure used. Taking stereo rotation capture as an example, the stationary 3-D wheel needs to have at least 6 equally sized sectors to reach a good percept of rotation. When using a sinusoidal grating as a stimulus (cf. second part in the video “Stereo Grating Rotation Phenomena”), it appears to rotate in the direction of the inducing dot texture, but somehow always flips back to its original orientation.

***Conclusions***. Both the SRSI and the SRCI demonstrate that in stereovision the percept of rotation, but not that of translation, is dominated by the luminance input. Since one does not even perceive the physically rotating 3-D wheel as rotating when using dynamic noise (flicker) to encode it, one has to conclude that stereo rotation detectors are either very weak or even missing. The failure of motion transparency when pitting luminance against depth motion resulting in both rotation standstill and capture illusions over the whole speed range adds evidence to the hypothesis that pure stereo vision is blind to rotation.

#### Stereo scaling standstill and motion capture illusions

“Scaling” denotes here two-dimensional expansion or contraction, i.e., size change or looming (Regan, 1986). To produce geometrically correct scaling and/or infinite texture motion, periodically repeating concentric rings where used for the 3-D rings and the random-dot texture (Figure 2B). The radii of the rings increase according to a geometric sequence. The *n*th term of a geometric sequence with an initial value of *a* and a common ratio of *r* is given by the formula: *a*_n_ = *ar*^*n* − 1^. Perceptually, looming is associated with motion in depth: away from the observer during contraction, toward the observer with expansion, even without supporting disparity changes.

During expansion, rings and/or circular periodical random-dot patterns are scaled up in size by increasing the scaling factor from *a* to *ar* or to *ar*^k^, where *k* is a positive integer. Then the initial ring's display is restored, and the rings expand again. The outermost and the innermost rings are masked to achieve a percept of continuous expansion. A similar scheme is used for contraction. In the video “Stereo Scaling Illusions,” it takes 5 s for the rings to travel from the initial to the third outermost position and the same time to reach the outmost position again corresponding to a temporal frequency of 0.40 Hz. To achieve this, the length and width of the rings' actual depth map used had to decrease/increase during contraction/expansion within 1 s to 85 or 117.6% of their starting size. But despite these facts, the rings appear not to contract/expand at all (“*Stereo Scaling Standstill Illusion”*) when the overlying random-dot pattern is not co-contracting/co-expanding. There is a sequence in the video, where yellow markers attached to the rings reveal their physical expansion or contraction. Note that the co-motion of the markers does not lead to a percept of expanding/contracting rings as they would with a flickering random dot pattern (not shown in the video).

If the 3-D rings remain physically stationary and the overlying random-dot pattern is expanding, one perceives the ring as expanding (“*Stereo Scaling Capture Illusion*”) and moving in depth toward the observer. This percept even remains in the presence of stationary yellow markers. Similarly, if the random-dot pattern is contracting, the rings appear to contract with the pattern moving in depth away from the observer even as the horizontal disparity of the rings remains constant.

***Conclusions***. Without supporting luminance motion input, stereo-vision is blind to size changes of a 3-D structure. The percept of motion in depth is dominated by luminance information.

#### Stereo shearing standstill and motion capture illusions

Shearing motion was implemented in the stereo Silverlight application following the definition by Koenderink (1986) for *pure shear*: “a contraction in one axis and an expansion in the orthogonal direction, such that area is conserved.” Pure shear has a common center of expansion and contraction. Two-dimensional pure shear has to be distinguished from one-dimensional shearing or relative motion (Golomb et al., 1985). As mentioned above, one-dimensional horizontal shearing of the random-dot pattern produces all the moving disparity gradients shown in our stereo videos.

In the accompanying video “Stereo Shearing Illusions” a sector wheel (Figure 2C) and a pattern of concentric rings (Figure 2D) was used as a 3-D structure. In the first half of a shearing cycle of 0.2 Hz the width of the rings are increasing to 120% of their smallest extent and their height decreasing from 120 to 100% of their smallest extent, in the second half their width is decreasing and their length increasing. With shearing sectors, it is difficult to get a percept of shearing motion at all (“*Stereo Shearing Standstill Illusion*”); with the rings one perceives residual, jerky shearing. The perceived shearing amplitude of the rings however appears smaller than the one indicated by the yellow makers or the one experienced during joint shearing of dots and 3-D rings. However, here we have a clear exception from the general rule that stereopsis is blind for all kind of complex motion. Depth perception makes use of vertical disparity shearing (Kaneko and Howard, 1997), i.e., it depends on finding slightly peripheral horizontal disparity gradients as contained in the concentric rings, but not in the sectors. Presumably, the information of the one-dimensional vertical shearing receptors is relayed to common complex motion detectors.

***Conclusion***. Without collaborating luminance shearing, stereo-vision is almost blind for shearing of a tracked 3-D structure. When the 3-D structure is made out of concentric ridges, position changes of the ridges can still be perceived resulting in a slowed-down, jerky shearing motion. Otherwise, the failure of motion transparency when pitting luminance against depth motion as evidenced by motion standstill and capture illusions for stereo rotation, expansions/contraction is in accordance with the hypothesis that pure stereo vision without help from the luminance system is blind for complex motion. Furthermore, even when using a dynamic texture to encode the 3-D structure, one never has the percept of a smoothly rotating, expanding/contracting, or shearing motion.

#### Stereo spiraling motion standstill and motion capture illusions

Spiraling motion is a combination of the two basic optic flow components, scaling and rotation. Our video “Stereo Spiraling Illusions” uses logarithmic spirals (Figure 2E). In parametric form, the formulas for a logarithmic right-handed spiral are: *x*(*t*) = *ae*^*bt*^ cos(*t*) and *y*(*t*) = *ae*^*bt*^ sin(*t*), with *a* and *b* being arbitrary real constants and *t* being an arbitrary rational value^1^. Note that logarithmic spiral have some uncommon features such that the scaling transformation also results in the spiral's rotation and rotation in the spiral's expansion or contraction. When the encoding random-dot pattern is stationary, the physically rotating spirals are perceived as stationary, even with the yellow markers turned on. With a clockwise rotating and contracting dot pattern, physically stationary spirals are perceived as spiraling away from the observer even as the disparity of the 3-D wheel remains the same.

***Conclusions***. 2-D luminance input produces a vivid 3-D motion percept even in the absence of corresponding disparity changes. Spiraling motion is a combination of two basic components of complex motion. As expected, the motion (or lack thereof) the random dot luminance texture determines how the depth motion is perceived.

### Color complex motion illusions

#### Methods

Several Silverlight 2 applications of my own making where used to search for the presumed color complex motion illusions. Once the motion ranges where such illusions would occur were roughly established, videos demonstrating color complex motion illusions were prepared with Adobe Premiere Pro CS5 taking advantage of its video motion effects for rotation and scaling or applying real dynamic random-dot textures. Using Magix Video deluxe 2014, the Windows Media videos were converted into web compatible MP4-format (resolution 1280 × 720 pixels, 25 frames/s).

**Figure 4:**
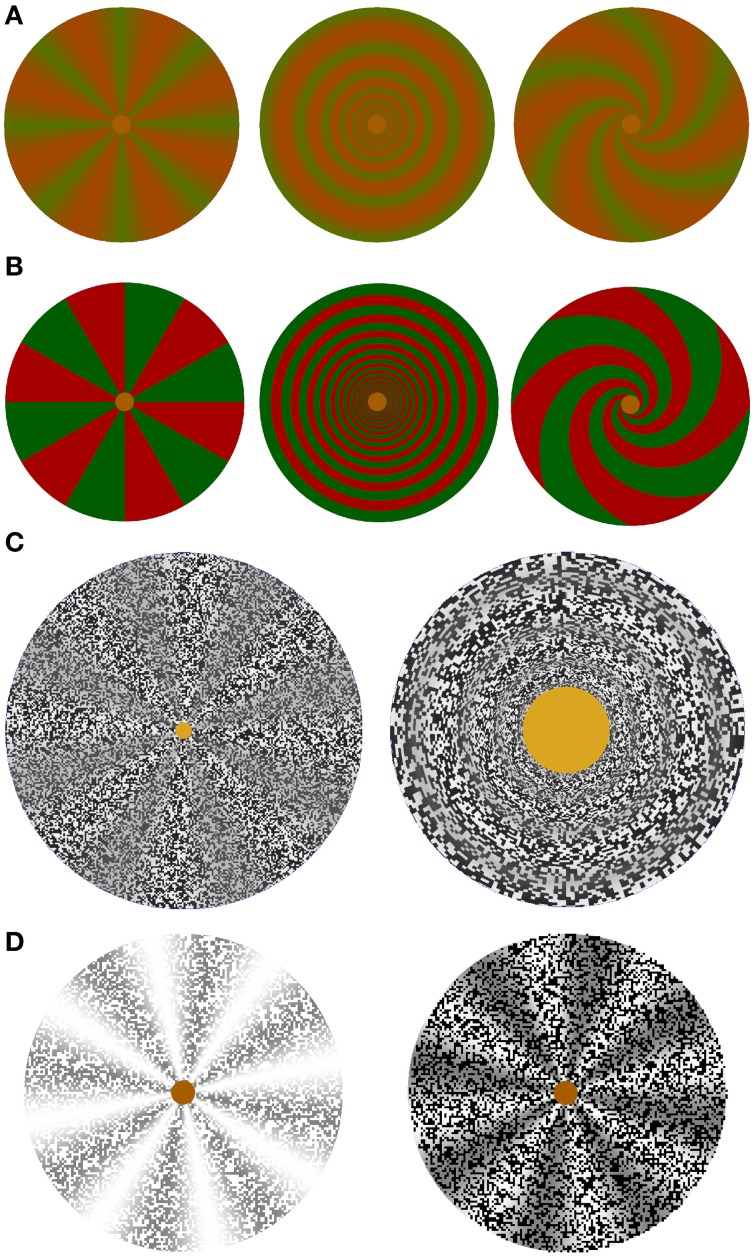
**Stimulus elements used to study color, color border, and contrast-modulated complex motion illusions**. **(A)** Isoluminant color stimuli with a smooth transition between green and red. Covered with a random dot pattern, they were used in the videos of color rotation, scaling or shearing and spiraling motion standstill and capture illusions demonstrating motion and complex motion blindness at low and complex motion blindness only at medium to high speeds. **(B)** Isoluminant color stimuli with a sharp border between red and green. Covered with a random dot pattern, color border standstill and capture can be observed only at very low speeds. **(C)** Screenshots of the random-dot texture-modulated wheel with 8 sectors on the left, which is used to study texture contrast modulated rotation. The texture-contrast modulated rings on the right are used to study scaling motion. Complex motion standstill and capture can be observed only at very low speeds. **(D)** Luminance stimulus shown with a white and with a black random-dot luminance mask used to study the influence of a luminance mask on rotations of a luminance stimulus. Luminance standstill and capture effects can be observed only at very low speeds.

The color in most of the accompanying videos (cf. Figure 4) changes sinusoidally from green to red (Cavanagh et al., 1984; Lu et al., 1999b). In other videos square-wave color stimuli are shown containing just two isoluminant red and green colors with a sharp border between them. The strength of color motion standstill and capture illusions depends on achieving isoluminance of the stimulus colors on the display, where the videos are shown. The setting we used to make the videos proved satisfying on many different thin-film-transistor liquid-crystal display (TFT LCD) devices with the exception of one laptop, where we had to tweak the video players color and luminance options to reach near isoluminance. For those readers who fail to achieve satisfactory isoluminance with their video player and therefore need to change the color setting, or those who wish to play with stimulus parameters, a Silverlight application demonstrating color complex motion illusions can be downloaded from the Figshare web site using the following URL: http://dx.doi.org/10.6084/m9.figshare.1149986.

#### Color translation standstill and motion capture

Only a few studies on color motion used rotation (Cavanagh, 1992; Seiffert and Cavanagh, 1999), the others used translation. Here we compare the perceived velocities of a translating sinusoidal and a square wave color grating. The accompanying video “Color Translation Illusions” shows in the upper half a sinusoidal and in the lower half a square-wave red and green grating moving back and forth at a speed of 16.3 pixels/sec (~0.5°/s). The upper sinusoidal grating conforms to a “*pure color*” stimulus with a spatial frequency of ~0.5 Hz/° while the lower square wave grating (“*color border*”) shows significant chromatic aberration artifacts at the color borders.

In the second scene the moving gratings are covered with a stationary random-dot mask with half of its pixels black and half transparent. The upper sinusoidal grating appears to be completely motionless (*color translation standstill*) while the lower square wave grating is still moving, giving an example of *motion transparency*. Closer inspection of the left or right window border reveals the upper grating's translation. For a short period, yellow markers (upper display) and black contours (lower display) appear which move with the gratings revealing their physical motion. In the third scene, the gratings and the dot textures move in synchrony. In the fourth scene, both gratings are stationary; the random-dot textures move back and forth at 16.3 pixels/s (~0.5°/s). In the upper display the percept is that of a moving color grating (*color translation capture*), in the lower display, one perceives dots sliding over the color bars. Yellow markers or black contours reveal that in fact the dots are sliding over stationary gratings.

In the second half the speed of the gratings and/or the dot texture is reduced to ¼ of the previous speed (~0.125°/s). Given that green and red are nearly isoluminant on an observer's monitor, one should perceive motion standstill and motion capture of the lower, red, and green square wave grating as well.

***Conclusions***. The static random dot pattern is able to induce color standstill in translating color stimuli at low speeds (~0.5°/s for a pure color and ~0.125°/s for a color border stimulus). A translating random dot pattern can “capture” the underlying color stimulus in a similar speed range. These findings confirm the well-known failure of color vision to perceive the motion of slowly moving large isoluminant stimuli (Ramachandran and Gregory, 1978; Cropper, 2005).

#### Color complex motion illusions

Color translation standstill and translation capture are phenomena that occur only at relatively low speeds. Since during rotation the speeds of the retinal slip increase linearly with the distance from the turning point, chances are that a rotation detector will sample from local motion detectors with a supra-threshold motion input. Alternatively a putative rotation detector may detect orientation changes and thus not depend on local motion detection at all (Benton et al., 2007; Lagacé-Nadon et al., 2009). Similar considerations are valid for scaling motion detectors, where the speed of the retinal slip increases as a power function of the distance to the center of expansion/contraction. Secondly, while first order (linear) motion detection thresholds for isoluminant stimuli may be masked by luminance noise as shown above, the global motion detection threshold in an isoluminant random-dot kinematograms (Michna and Mullen, 2008) was not affected by luminance noise. Therefore, global chromatic motion information may be relayed to putative complex motion detectors even in the presence of a random-dot pattern luminance mask.

#### Color rotation standstill and capture

In the accompanying video “Color Rotation Illusions,” a color wheel made out of sectors whose colors change smoothly from green to red (cf. Figure 4A, leftmost stimulus), is turning at a speed of 6°/s, its direction is changing every 5 s. In the second scene, a stationary black and transparent random-dot pattern covers the turning color wheel. The color wheel appears as motion-less (*color rotation standstill*). In fact, we perceive no motion at all, even when looking at the wheel's rim. At the beginning, a red arrow-head indicates the physical rotation of the wheel. Even with holes in the random-dot pattern, the color wheel's rotation appears still suppressed in the covered regions. At high rotational speeds (24°/s in the video), the wheel still appears as motion-less with central fixation, but appears to rotate readily when looking more peripherally. At much higher speeds (≥60°/s), the wheel was perceived as either rotating or as rotating not smoothly, when fixating the center of rotation (not shown in the video). The upper limit for perceiving a rapidly rotating pure color wheel as stationary or rotating in jerks depends on factors such as the quality of fixation at the center of rotation with the gaze axis perpendicular to the screen, the use of colors which appear isoluminant to the observer, the maximal color contrast used and a sufficiently high frame rate of the monitor. With an optimal setting, I was not able to perceive color rotation even at a rotational speed of 60°/s.

In the next scene, wheel and dots turn in synchrony; in the following sequence, the dot pattern is turning at 6°/s and the color wheel is stopped. In the presence of a rotating random-dot pattern, a physically stationary color wheel is perceived as rotating (*color rotation capture*). With good central fixation, motion capture can still be perceived at an angular dot pattern speed of 24°/s or above. Sometimes, a paradoxical percept may arise in which the wheel appears to rotate but does not change its orientation.

The latter scenes in the video demonstrate that color rotation standstill can also be obtained using a dynamic (*flickering*) random-dot pattern, where the random-dot texture is changed from frame to frame. While yellow markers rotating underneath a static random-dot texture appear not to capture the color wheel's motion, yellow markers rotating underneath a dynamic luminance mask now seem to capture it, i.e., the wheel is perceived as co-rotating with the markers. Note that the flickering dots also appear to co-rotate with the markers.

A rotating color sinusoidal grid may be perceived as rotating, albeit very slowly with a jerky motion (cf. first part of the video “Color Grating Rotation Illusion”). When the random-dot pattern is rotating, the color grid appears to rotate in the direction of the inducing dot texture, but somehow always flips back to its original orientation.

***Conclusion***. If the hypothesis is correct that the position based, general purpose feature motion tracking system is unable to detect complex motion such as rotation, I should be able to show not only stereoscopic, but color rotation motion standstill and capture illusions for the whole range of speed as well, where the feature tracking system is operative (low to medium speeds). This is in fact the case. In addition, when using dynamic noise, one is unable to perceive complex color rotation; a stationary chromatic wheel appears to co-rotate with rotating luminance blotches (markers).

#### Border color rotation standstill and capture

In the accompanying video “Color Border Rotation Illusions” a color wheel with sharp *color borders*, i.e., its sectors painted alternatively in isoluminant red or green, is used (cf. Figure 4B, leftmost stimulus). As discussed above, such a stimulus introduces luminance artifacts mostly due to chromatic aberration (Cavanagh and Anstis, 1991) and its higher chromatic contrast will increase its contrast gain (Hawken et al., 1994; Seiffert and Cavanagh, 1999). The “color border” stimulus is more likely to be processed by a second more velocity-sensitive mechanism, which detects motion of luminance pattern, isoluminant color, and contrast-modulated textures of high contrasts and speeds (Seiffert and Cavanagh, 1999). Therefore, the speed required to perceive motion transparency is much lower for the “color border” stimulus than for the “pure color” stimulus with smooth transitions between red and green. In fact, the rotational speed of the color border stimulus had to be set as low as 4°/s to get the perception of a motionless wheel in the presence of a stationary random-dot pattern. At medium angular speeds (12°/s), the perceived color wheel's speed is slower than the physical speed (not shown in the video). At high angular speeds (24°/s) of the wheel, the perceived speed appears to be close to the real speed. Motion capture could only be demonstrated for very low rotation speeds of the inducing dots (4°/s).

A dynamic random-dot pattern (flicker) did not impair the rotation perception at all (angular wheel speed 4°/s).

***Conclusion***. Given a high contrast color stimulus moving at rotational speeds well above 4°/sec, chances are that its motion is detected by a general purpose velocity-sensitive mechanism (Gegenfurtner and Hawken, 1996; Seiffert and Cavanagh, 1998, 1999) which has an output to a higher order complex motion detecting system. This is in accordance with the hypothesis that the deficit of complex color motion is restricted to the operative range of the salient feature tracking system.

#### Color scaling standstill and capture

In the video “Color Scaling Illusions,” concentric isoluminant rings, whose colors change from red to green (cf. Figure 4A, middle stimulus) are contracting for 5 s, than expanding for the next 5 s, thereby increasing their scale to 146% of their smallest value. Than a stationary luminance mask with black random dots is added leading to a percept of a *color scaling standstill*. Yellow markers appear for a short period to show the physical scaling motion of the rings.

After a sequence, where dots and rings contract/expand in synchrony, a video section is shown, where only the random-dot pattern is alternatively contracting or expanding, while the color rings do not change in size. However, perceptually the rings appear to shrink and grow together with the dot pattern (*color scaling capture*).

When using color border rings painted alternatively in isoluminant green and red (cf. Figure 4B, middle stimulus), the scaling speeds of the rings or dot texture had to be reduced (≤121% change in 5 s) to perceive border color scaling standstill or scaling capture (not shown in the video).

***Conclusions***. Showing color scaling standstill and capture illusions for pure color stimuli only adds evidence to the hypothesis, that the color system is blind to complex motion within the operating range of the position-based feature tracking system.

#### Color shearing standstill and capture

An isoluminant sector wheel and an array of concentric rings are used to demonstrate pure shearing (cf. video “Color Shearing Illusions”), i.e., expanding within 5 s to 146% of their smallest extent along the horizontal axis and contracting from 146 to 100% along the vertical axis or vice versa. During the *color shearing standstill* conditions only the sectors or rings are shearing while the random-dot patterns are stationary; the arising percept is that of motionless sectors or rings (here is a difference to stereopsis, motion where 3-D rings, but not 3-D sectors were still perceived as jerkily shearing when their depth was encoded using a stationary random-dot pattern).

During joint shearing color stimuli and random dots are shearing in synchrony, during the *color shearing capture* condition, the rings or sectors are stationary, while only the dots patterns are shearing. However, the rings or sectors appear as shearing too. Even in the presence of stationary yellow markers it is very difficult for an observer to assure that they are not shearing at all.

***Conclusions***. The pure color system is also blind to shearing color motion.

#### Color spiraling standstill and capture

In the accompanying video “Color Spiraling Motion Illusions,” wheels made of 12 logarithmic spirals, whose color changes smoothly from red to green (cf. Figure 4A, rightmost stimulus), are used to demonstrate spiraling motion standstill and capture. In the standstill presentation, the spirals to the left are scaled up in size up to 350% of their original size, which results in about the same motion effect as a rotation of 90° for the spirals on the right side. After 5 s, the spirals shrink or rotate backwards. In the motion capture section, the random-dot texture is scaled up to 146% of its original size while rotating in the same time period of 5 s at 9°/s.

***Conclusions***. The color system is also blind to the combination of two basic complex motion elements such as rotation and expansion/contraction.

### Additional complex motion illusions

Overlying a grille made out of a random-dot pattern on a moving visual stimulus increases its speed threshold for perceiving motion. Here I demonstrate the occurrence of (complex) motion standstill and capture for second and first order stimuli. Note that these speeds are well below the speeds at which I demonstrated complex motion standstill and capture for pure stereo or color stimuli.

#### Texture-contrast modulated complex motion

*Texture-contrast modulated complex motion* (Lu and Sperling, 1995b) was examined as the classical example of a second-order motion system. It uses, like the stereo system, a richly structured texture as a carrier to encode the moving stimulus. The contrast of the carrier random-dot texture was varied according to the brightness intensity of a given figure such as the sector wheel or the concentric rings used in the other experiments (cf. Figure 4C). In the accompanying video “Contrast Modulated Motion Illusions,” texture-contrast modulated complex motion standstill and capture for rotation and scaling are demonstrated. The rotational speed of the contrast wheel had to be reduced to 4°/s to achieve a less than perfect percept of a motionless contrast wheel. At higher rotational speeds, the wheel is perceived as rotating, albeit at a lower than its physical angular speed (not shown in the video).

With a stationary contrast encoded wheel and a rotating contrast carrier texture, a percept of rotation capture could only be obtained at angular speeds up to 4°/s. In the scaling demonstration, the scale of the contrast rings or the random-dot pattern changed between 100 and 121% of its original size for periods of 5 s; with this scaling speed one gets less than perfect percepts of scaling standstill or scaling capture.

***Conclusions***. For texture-contrast modulated random-dot patterns, rotation and scaling standstill or capture illusions can be demonstrated at very low speeds of either the contrast encoded stimulus or the random dot pattern, but already at speeds higher than 6°/s motion transparency is perceived. At these speeds, motion transparency fails for pure color or stereo stimuli. To demonstrate that a given motion system is motion blind, motion standstill and capture illusions have to be demonstrated over the whole operational speed range of the examined motion subsystem. The texture-contrast modulated complex motion system is well able to process complex motion.

#### Luminance rotation illusions

Livingstone and Hubel (1987) reported that the perceived rotation speed of a low luminance stimulus was four times slower than that of a high a high contrast stimulus, whereas in more controlled experiments Vaziri-Pashkam and Cavanagh (2008) found an overestimation of the rotation speed at low luminance, a good reason to look for the effects of random-dot luminance masks on rotating luminance stimuli. Albright and Stoner (1995) have elaborated rules about depth-ordering of (moving) plaids and luminance configurations compatible with transparency or occlusion. Therefore, not only a black-transparent random-dot texture (cf. Figure 4D, right side), but also a white-transparent dot texture (cf. Figure 4D, left side) were used (see accompanying video “Luminance rotation illusions”). The white dots are more efficient to achieve the percept of an almost motion-less wheel, which was physically slowly rotating (4°/s) back and forth. Note that when replacing the black with the white dots, the 3-D appearance of the sector wheel is lost.

Slowly rotating (4°/s) white dots are also more efficient to evoke the percept of a rotating luminance wheel with a physically stationary wheel (luminance rotation capture).

At higher rotation speeds of the luminance wheel covered by a stationary white-transparent random-dot mask is perceived as rotating. The perceived wheel speed is close to its physical speed of 24°/s.

***Conclusions***. The maximal speeds at which motion standstill and capture can be shown with first or second order stimuli (luminance or texture-contrast modulated patterns) are much lower than the maximal speeds with third order stereo or color motion. Color stimuli with sharp borders have similar speed ranges as equivalent luminance stimuli overlaid with a white-transparent random dot pattern. These findings are in accordance with the hypothesis, that deficits to perceive complex motion are restricted to the operating range of the putative feature tracking system, which processes color stimuli with smooth transitions between their colors or stereoscopic stimuli only.

## Discussion

### Complex motion transparency and its failures with stereoscopic and color stimuli

We tested complex motion transparency by pitting a luminance pattern against other luminance, contrast-modulated, stereoscopic, or color stimuli with sharp (“color border”) or smooth transitions (“pure color”) between red and green. Either the overlying luminance pattern or the underlying stimulus was rotating contracting, expanding, or shearing. As outlined in the introduction, a failure of motion transparency resulting in motion standstill or motion capture over a wide range of speeds would indicate an absence or a suppression of the motion system studied.

While looking for the minimal velocity difference between two stimuli which had to be reached to perceive motion transparency, different perceptual outcomes between these stimulus combinations where found. For either *stereoscopic* or *pure color* stimuli paired with a luminance pattern, the threshold for complex motion transparency could not be reached even at medium to high velocities differences (e.g., 12°/s for rotation, cf. Table 1). In either combination of a stereo or a pure color stimuli paired with a luminance pattern, the *complex motion percept was dominated by the luminance pattern* leading either to the percept of *complex motion standstill*, when the luminance pattern was stationary, or to the percept of complex *motion capture*, when it was moving.

When replacing the static random dot pattern placed over physically rotating, scaling or shearing stereoscopic or pure color stimuli with a *dynamic* (*flickering*) pattern, they appeared still as motion-less, however, when yellow luminance markers moving in synchrony with the ridges of the stereoscopic stimulus or the red parts of the color stimulus were added, they were perceived as moving smoothly with the markers.

Taken together, these findings are in accordance with the hypothesis that both the pure color and the stereo motion perception are blind for complex motion. In both systems there are exceptions to this general rule which are discussed below.

#### Complex motion transparency failures with stereoscopic stimuli

For the combination of a *stereo and a luminance pattern*, one is able to perceive local (*translational*) stereoscopic motion (e.g., the motion at a spoke's end) even at very low speeds of the 3-D structure (6°/s). I demonstrate in one of the introductory videos that the perceived speed of a translating sinusoidal depth grating is much lower than the perceived speed of a luminance pattern moving at physically the same speed. Chang (1990) studied translation stereo-motion using a similar technique as mine. He reported a failure of motion transparency with a dominance of the depth-encoding random-dot luminance pattern, when the luminance pattern was moving at a right angle or opposite to the encoded depth pattern.

For *complex motion* we never reached the threshold to get a percept of complex motion transparency within the range of motions where the 3D-structure remained clearly visible (Dürsteler and Lorincz, 2014). For perfect complex motion standstill or motion capture to occur, certain form constraints must be met: e.g., for rotations, the 3-D structure should be radially symmetric with a minimal number of repeating sectors. With less perfect forms such as a rotating sinusoidal grating, a residual, somewhat shaky rotation is perceived reminiscent to the description of motion blindness in humans (Zihl et al., 1983). A possible exception to this general rule is shown here for pure shearing of circumferential, but not radial, disparity gradients. It appears that the disparity system without help from other visual sub-modalities is blind to (short-range) rotation, scaling and partly to shearing. The main task of the disparity system is to exploit the advantage of having two eyes by providing us with information about depth, inclination, and slant (Howard and Kaneko, 1994; Kaneko and Howard, 1996, 1997). The simultaneous processing of complex depth motion could interfere with these tasks. Since in everyday life the processing of complex motion is performed so well by other visual sub-modalities such as the luminance system, the disparity system does not need to participate.

#### Complex motion transparency failures with color stimuli

With slowly rotating/expanding/shearing *pure color stimuli*, motion transparency fails for slow *local (translational) motion*. The question is why one fails to perceive simple color motion. At low to moderate spatial frequencies (<1 Hz) isoluminant color stimuli are perceived to move more slowly than luminance stimuli moving at the same speed and the same effective contrast (Cavanagh et al., 1984; Hawken et al., 1994; Seiffert and Cavanagh, 1999). For isoluminant color stimuli, the contrast threshold for pattern detection is much lower than the threshold for motion detection, whereas they are the same for luminance stimuli (Lindsey and Teller, 1990; Derrington and Henning, 1993). Thus, one can detect low-contrast color stimuli without seeing their motion (Ramachandran and Gregory, 1978). In the absence of a chromatic first order motion mechanism (Yoshizawa et al., 2000), an observer may detect its motion by tracking it over time, but motion will be rather jerky (Mullen and Boulton, 1992). The presence of an additional static or dynamic luminance mask helps to achieve color motion standstill at low to medium speeds (Culham and Cavanagh, 1994; Thiele et al., 1999; Michna and Mullen, 2008). When rising the velocity difference (e.g., from 6 to 24°/s for rotation of the color sector wheel), the local (translational) motion of a single sub-component of the color stimulus such as a red spoke becomes clearly visible, whereas the perception of complex motion is dominated by the motion of the overlying luminance pattern resulting in either complex color motion standstill or capture illusions (cf. Ramachandran, 1987).

Next I am posing the question, why the deficit to perceive complex color motion is restricted to isoluminant stimuli with low spatial and temporal frequency content, whereas one perceives the complex motion isoluminant stimuli with high spatial and/or temporal frequency content (e.g., my color border stimuli) as well as that of luminance defined stimuli.

For low to moderate color contrasts and speeds, color motion perception depends on a position-tracking mechanism (Seiffert and Cavanagh, 1999) which may correspond to the third order motion mechanism of Lu et al. (1999a); Lu and Sperling (1995a). A second, velocity-sensitive mechanism, which detects isoluminant color, luminance pattern and texture modulated gratings alike, is operative for stimuli with a contrast above ten times the detection threshold or high speeds (Gegenfurtner and Hawken, 1996; Seiffert and Cavanagh, 1999).

Increasing the spatial frequency of a nominally isoluminant color stimulus increases the chromatic aberration of its retinal image thereby introducing significant luminance artifacts. To avoid artifacts due to chromatic aberration in the human eye, the spatial frequency of the color stimuli should be ≤0.5 Hz (Cavanagh and Anstis, 1991). Additional internal luminance contaminations arise during the internal processing of nominally isoluminant chromatic stimuli either due to different neuronal response latencies to opponent colors or due to different isoluminant points of neurons in the color motion pathways (for reviews see Cropper and Wuerger, 2005; Shevell and Kingdom, 2008). As we will discuss in more detail below, there seems to be a dichotomy in the cortical color pathways (Shapley and Hawken, 2011), where the single-opponent pathway processes low spatial frequency and the double-opponent pathway high spatial frequency color stimuli. The motion of color stimuli with smooth transitions between colors will be processed by the single-opponent color system, whose output goes into the position-tracking or third order motion-system. The motion of color stimuli with sharp borders between colors like in the “color border” examples below will in all likelihood be processed by the dual-opponent color system. It most probably feeds into the velocity-sensitive-based motion system mentioned above which is well equipped to analyze complex motion.

The failure of complex motion transparency for chromatic stimuli with a smooth transition between the opponent colors, but not for chromatic stimuli with a sharp transition between colors adds evidence for the existence of two different motion processing mechanisms in color vision: (i) A *position-based color motion system* which is unable to compete with locally conflicting motion from other visual sub-modalities, and which appears to be blind for rotating, scaling and shearing motion. This system may be identical with the third order feature tracking system of Lu and Sperling (2001). (ii) A *velocity-based color/luminance motion system* as described by Hawken et al. (1994) and Seiffert and Cavanagh (1999). It achieves motion transparency when competing with other visual sub-modalities and it feeds into a complex motion processing mechanism shared with the luminance system.

### Luminance contrast or texture-contrast modulated stimuli

In the two stimulus combinations, where a luminance pattern is pitted against a *luminance contrast modulated stimulus* or a *texture-contrast modulated stimulus*, a failure of motion transparency with a dominance of the random-dot pattern is observed only at very low velocity differences(e.g., 4°/s for rotations, cf. Table 1), resulting in complex motion standstill and capture. The maximal speeds at which we still perceive a solid standstill illusion are in a similar small range for luminance, color border, and texture-contrast modulated stimuli.

At higher speeds *motion transparency* is experienced, i.e., either the object is perceived as moving while the overlying semi-transparent random-dot texture appears as stationary or vice versa. Motion transparency could also be demonstrated when random dots and the luminance-contrast modulated stimulus were moving in different directions. It is important to realize that there are multiple complex motion processing mechanisms operating on a same part of the visual field in order to achieve complex motion transparency. These mechanisms may be shared between luminance, color border, and possibly texture-contrast modulated systems.

### Neuronal correlates of color and stereo-motion perception

The visual system uses different pathways to process local brightness, color, or disparity motion information at lower levels. We may note that starting from V1 all to way to the infra-temporal cortex, there appears to be a dichotomy in the color pathways according to the spatial frequency content of the color stimulus (Shapley and Hawken, 2011). In V1 of the macaque, the population spatial frequency tuning curve of color preferring neurons corresponds to a low-pass filter with a mean spatial frequency of ~0.5 Hz/°, whereas the spatial frequency tuning curves both of color-luminance neurons responding equally well to color and luminance, and luminance preferring neurons correspond to band-pass filters with peaks at ~2 Hz/° (Schluppeck and Engel, 2002; Johnson et al., 2004). A typical representative of a color preferring neuron is the non-oriented, single-opponent color cell responding best to large color blobs; a typical representative of a color luminance neuron is the oriented double-opponent color cells responding preferentially to color pattern, textures and boundaries (for a review see Shapley and Hawken, 2011). In the macaque monkey, the pathways for brightness, color, or disparity motion finally converge in area MT (Maunsell and Van Essen, 1983a,b; DeAngelis et al., 1998; DeAngelis and Newsome, 1999; Seidemann et al., 1999; DeAngelis and Uka, 2003; Liu and Newsome, 2003; Barberini et al., 2005).

Within their receptive fields, MT cells react to moving luminance and/or *color* stimuli (Thiele et al., 1999; Barberini et al., 2005) and/or *disparity* gradients (DeAngelis and Newsome, 1999; DeAngelis and Uka, 2003). MT neurons are organized into columns according to disparity (DeAngelis and Newsome, 1999), speed (Liu and Newsome, 2003) and depth tuning based on motion parallax (Nadler et al., 2013). There is evidence from an fMRI-study that the human MT+ complex encodes three-dimensional motion by processing disparities or inter-ocular velocity differences changing over time (Rokers et al., 2009). While some MT cells responded well to moving equiluminant chromatic grating, this response could easily be suppressed by a luminance contrast grating moving in the opposite direction (Thiele et al., 1999). MT cells react to local, but are unaffected by global, translational motion (Hedges et al., 2011).

The anatomical structures, which according to current experimental evidence are involved in the detection of motion transparency, are the medial temporal region (MT) and medial superior temporal region (MST) in the macaque monkey (Qian and Andersen, 1994).

The output of neurons with small receptive fields from V1 up to MT is integrated by specialized large field motion mechanisms known to operate in the extra-striate regions upstream from MT. The first neurons in the visual pathways which respond to *radial, circular and spiral motion*, have been found in the dorsal region of the medial superior temporal region (MSTd) of the macaque monkey (Tanaka and Saito, 1989; Duffy and Wurtz, 1991a,b; Graziano et al., 1994; Geesaman and Andersen, 1996; Takahashi et al., 2007; Mineault et al., 2012) and in the V5/MT+ complex of the human (Culham et al., 2001; Dukelow et al., 2001). Humans with lesions involving the human homolog of MST have profound difficulties navigating in their surroundings (Vaina, 1998). It could well be that the MST neurons, which analyze optic flow, have their input mainly from MT cells within speed clusters and fewer inputs from MT-cells deep within disparity clusters which would explain why the disparity system is so poor in detecting complex motion. No study however has so far evidence for detectors that combine complex motion and disparity or color processing.

### Conclusions

Within the operating ranges of the putative feature tracking motion system in stereo and color vision, we did not find evidence for the existence of stereo-motion or pure color-motion inputs to the complex motion system, as we never observed smooth complex motion of the stereoscopic or the pure color stimulus without the capturing effect of luminance defined texture or marker, even when using a dynamic random-dot stereogram or color mask. Seemingly even the hypothetical third order motion system based on saliency (Lu and Sperling, 2001) could not detect the complex motion of our stereo or pure color stimuli rich in salient features. If the detection of complex motion depends exclusively on inputs from a general-purpose first-order system, it is likely to give rise to “complex motion standstill” or “complex motion capture illusions,” whenever one is looking at a scene with moving objects with an incongruence between the complex motion of luminance (texture or markers) and the stereoscopic or chromatic motion. However, such situations are very unlikely to occur in daily life.

Regarding color vision, additional evidence was found for the existence of two separate color motion systems (Hawken et al., 1994; Seiffert and Cavanagh, 1999): (i) a feature tracking system for isoluminant low frequency chromatic stimuli with smooth transitions between their colors. (ii) A velocity based first order system for stimuli with sharp transitions between their colors and/or moving at high speeds. This system is also used to process luminance motion. It feeds into a common complex motion processing system. The existence of two color motion systems for pure color or color-border and luminance motion fits well into the idea of a dichotomy in the color system, of which V1 single or dual-opponent neurons are a part (Shapley and Hawken, 2011).

### Conflict of interest statement

The author declares that the research was conducted in the absence of any commercial or financial relationships that could be construed as a potential conflict of interest.

## Footnotes

^1^Wikipedia. (2014, 03-04-2014). Logarithmic spiral. Wikipedia, Available online at: en.wikipedia.org/wiki/Logarithmic_spiral

## Appendix

**Table:** **List of accompanying videos (the videos can be found on http://dx.doi.org/10.6084/m9.figshare.1014283**).

**Filename**	**Content**
MotionTransparency&FlickerLumLum.mp4	Demonstration of motion transparency with a luminance random dot pattern and a luminance grating
MotionTransparency&FlickerLumColor.mp4	Motion transparency with a luminance random dot pattern and a color grating
MotionTransparency&FlickerLumDepth.mp4	Motion transparency with a luminance random dot pattern and a stereoscopic grating
Stereo Rotation Illusions.mp4	Stereo rotation, rotation standstill & capture
Stereo Grating Rotation Phenomena.mp4	Demonstration of stereo complex motion blindness using a rotating 3-D sinusoidal grating
Stereo Scaling Illusions.mp4	Stereo contraction/expansion standstill & capture
Stereo Shearing Illusions.mp4	Stereo shearing standstill & capture with rings or sectors
Stereo Spiraling Motion Illusions.mp4	Scaling and rotation of logarithmic spirals, stereo spiraling motion standstill and capture
Color Translation Illusions.mp4	Translation standstill and capture with smooth and sharp color borders
Color Rotation Illusions.mp4	Color rotation, rotation standstill & capture with smooth color transitions
Color Grating Rotation Phenomena.mp4	Demonstration of motion, but not position blindness with a color grating
Color Scaling Illusions.mp4	Color contraction/expansion standstill & capture with smooth transitions
Color Shearing IIusions.mp4	Color shearing standstill & capture with smooth color transitions
Color Spiraling Motion Illusions.mp4	Color spiraling motion standstill and capture with smooth color transitions
Color Border Rotation Illusions.mp4	Color border standstill and capture at low, but not at medium angular speeds
Contrast Modulated Motion Illusions.mp4	Rotation and scaling standstill or capture with a texture-contrast modulated slowly moving stimulus
Luminance Rotation Illusions.mp4	Rotation standstill and capture illusion with a slowly moving luminance contrast modulated stimulus
